# Supplementary material for: The causal relationship between the human gut microbiota and pyogenic arthritis: a Mendelian randomization study
Source: Front Cell Infect Microbiol. 2024 Nov 26;14:1452480. doi: 10.3389/fcimb.2024.1452480 (PMC11629706; doi:10.3389/fcimb.2024.1452480)
Supplement: Supplementary file 1 [file DataSheet1.zip › mendelian test/ebi-a-GCST90027679.csv_leave_one_out.pdf]

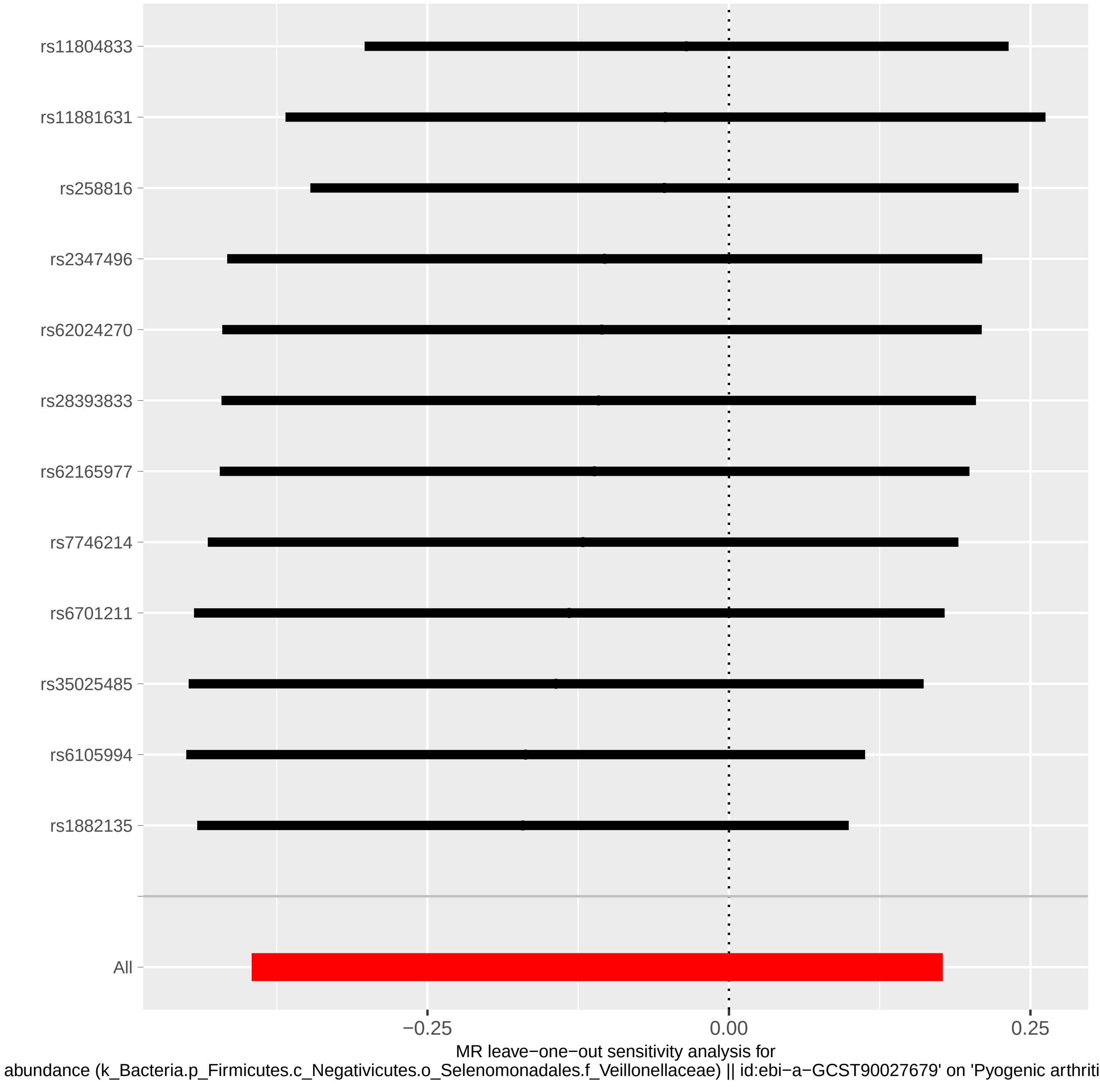
rs11804833
rs11881631
rs258816
rs2347496
rs62024270
rs28393833
rs62165977
rs7746214
rs6701211
rs35025485
rs6105994
rs1882135
All
−0.25
0.00
0.25
MR leave−one−out sensitivity analysis for
abundance (k_Bacteria.p_Firmicutes.c_Negativicutes.o_Selenomonadales.f_Veillonellaceae) || id:ebi−a−GCST90027679' on 'Pyogenic arthriti
